# A mathematical model reveals the influence of population heterogeneity on herd immunity to SARS-CoV-2

**DOI:** 10.1126/science.abc6810

**Published:** 2020-06-23

**Authors:** Tom Britton, Frank Ball, Pieter Trapman

**Affiliations:** 1 Department of Mathematics, Stockholm University, Stockholm, Sweden.; 2 School of Mathematical Sciences, University of Nottingham, Nottingham, UK.

## Abstract

Despite various levels of preventive measures, in 2020, many countries have suffered severely from the coronavirus disease 2019 (COVID-19) pandemic caused by the severe acute respiratory syndrome coronavirus 2 (SARS-CoV-2) virus. Using a model, we show that population heterogeneity can affect disease-induced immunity considerably because the proportion of infected individuals in groups with the highest contact rates is greater than that in groups with low contact rates. We estimate that if *R*_0_ = 2.5 in an age-structured community with mixing rates fitted to social activity, then the disease-induced herd immunity level can be ~43%, which is substantially less than the classical herd immunity level of 60% obtained through homogeneous immunization of the population. Our estimates should be interpreted as an illustration of how population heterogeneity affects herd immunity rather than as an exact value or even a best estimate.

## Heterogeneity and herd immunity

In response to severe acute respiratory syndrome coronavirus 2 (SARS-CoV-2), some politicians have been keen to exploit the idea of achieving herd immunity. Countering this possibility are estimates derived from work on historical vaccination studies, which suggest that herd immunity may only be achieved at an unacceptable cost of lives. Because human populations are far from homogeneous, Britton *et al.* show that by introducing age and activity heterogeneities into population models for SARS-CoV-2, herd immunity can be achieved at a population-wide infection rate of ∼40%, considerably lower than previous estimates. This shift is because transmission and immunity are concentrated among the most active members of a population, who are often younger and less vulnerable. If nonpharmaceutical interventions are very strict, no herd immunity is achieved, and infections will then resurge if they are eased too quickly.

*Science*, this issue p. 846

## Teaser

Human population heterogeneities bring down estimates for herd immunity.

Severe acute respiratory syndrome coronavirus 2 (SARS-CoV-2) has spread globally despite the many different preventive measures that have been put in place to reduce transmission. Some countries aimed for suppression by extreme quarantine measures (lockdown) and others aimed for mitigation by slowing the spread using certain preventive measures in combination with protection of the vulnerable (*1*). An important question for both policies has been when to lift some or all of the restrictions. A closely related question is if and when herd immunity can be achieved. Herd immunity is defined as a level of population immunity at which disease spreading will decline and stop even after all preventive measures have been relaxed. If all preventive measures are relaxed when the immunity level from infection is below the herd immunity level, then a second wave of infection may start once restrictions are lifted.

By 1 May 2020, some regions and countries reached high estimates for the population immunity level; for example, 26% of the population was infected (with a large confidence interval) in the metropolitan Stockholm region, as shown by a mathematical model (*2*). At the same time, population studies in Spain showed that in the second half of May 2020, >10% of the population of Madrid had antibodies for coronavirus disease 2019 (COVID-19) (*3*). It is debatable whether (classical) herd immunity for COVID-19, which is believed to lie between 50 and 75%, can be achieved without unacceptably high case fatality rates (*4*–*6*).

The definition of classical herd immunity originates from mathematical models for the impact of vaccination. The classical herd immunity level *h*_C_ is defined as *h*_C_ = 1 – 1/*R*_0_, where *R*_0_ is the basic reproduction number, defined as the average number of new infections caused by a typical infected individual during the early stage of an outbreak in a fully susceptible population (*7*). Thus, if a fraction *v* is vaccinated (with a vaccine giving 100% immunity) and vaccinees are selected uniformly in the community, then the new reproduction number is *R_v_* = (1 – *v*)*R*_0_. From this, the critical vaccination coverage *v_c_* = 1 – 1/*R*. So, if at least this fraction is vaccinated and hence immune, the community has reached herd immunity because *R_v_* ≤ 1 and no outbreak can take place. If the vaccine is not perfect but instead reduces susceptibility by a fraction *E* (so *E* = 1 corresponds to 100% efficacy), then the critical vaccination coverage is given by *v_c_* = *E*^–1^(1 – 1/*R*_0_) (*7*), implying that a bigger fraction needs to be vaccinated if the vaccine is not perfect.

No realistic model will depict human populations as homogeneous; there are many heterogeneities in human societies that will influence virus transmission. Here, we use a model to illustrate how population heterogeneity can cause substantial heterogeneity among the people infected during the course of an infectious disease outbreak, with consequent impact on the herd immunity level and the performance of exit policies aimed at minimizing the risk of future infection spikes.

One of the simplest of all epidemic models is to assume a homogeneously mixing population in which all individuals are equally susceptible and equally infectious if they become infected. Before becoming infectious, infected individuals first go through a latent/exposed period, i.e., the susceptible-exposed-infected-recovered (SEIR) model (*7*). The basic reproduction number *R*_0_ denotes the average number of infectious contacts that an infected individual has before recovering and becoming immune (or dying). An infectious contact is defined as one close enough to infect the other individual if this individual is susceptible (contacts with already infected individuals have no effect).

To this simple model, we add two important features known to play an important role in disease spreading (the model is described in full detail in the supplementary materials). The first is to include age structure by dividing the community into different age cohorts with heterogeneous mixing between them. We categorized a community into six age groups and fit contact rates derived from an empirical study of social contacts (*8*) (see the supplementary materials for details on the community structure). The person-to-person infectious contact rate between two individuals depends on the age groups of both individuals. The average number of infectious contacts that an infected person in age group *i* has with individuals in (another or the same) age group *j* now equals *a_ij_*π*_j_*, where *a_ij_* reflects both how much an *i* individual has contact with a specific *j* individual. It also reflects the typical infectivity of *i* individuals and susceptibility of *j* individuals. The population fraction of individuals belonging to age cohort *j* is denoted by π*_j_*.

The second population structure element that we added to the simple model categorizes individuals according to their social activity level. A common way to do this is by means of network models [e.g., (*9*)]. Here, we take a simpler approach and categorize individuals into three different activity levels, which are arbitrary and chosen for illustration purposes: 50% of each age cohort have normal activity, 25% have low activity corresponding to half as many contacts compared with normal activity, and 25% have high activity corresponding to twice as many contacts as normal activity. By this we mean that, for example, a high-activity individual in age group *i* on average has 2*a_ij_π_j_*0.5*0.25 infectious contacts with low-activity individuals of age group *j*. The factor 2 comes from the infective having high activity, the factor 0.5 from the contacted person having low activity, and the factor 0.25 from low-activity individuals making up 25% of each age cohort. The basic reproduction number *R*_0_ for this model is given by the dominant eigenvalue of the (next-generation) matrix *M* having these elements as its entries. (*7*).

The final fractions of the different groups in the population becoming infected in the epidemic are obtained by solving a set of equations (the final size equations are provided in the supplementary materials). To be able to say something about the time evolution of the epidemic, we assume a classical SEIR epidemic model. More precisely, we assume that individuals who get infected are initially latent for a mean of 3 days, followed by an infectious period of a mean of 4 days, thus approximately mimicking the situation for COVID-19 [e.g., (*1*)]. During the infectious period, an individual makes infectious contacts at rates such that the mean numbers of infectious contacts agree with those of the next-generation matrix *M*.

In the model, we assume that the basic reproduction number satisfies *R*_0_ = 2.5 (a few other values are also evaluated) and that the epidemic is initiated with a small fraction of infectious individuals on 15 February. On 15 March, when the fraction infected is still small, preventive measures are implemented such that all averages in the next-generation matrix are scaled by the same factor α < 1, so the next-generation matrix becomes α*M*. Consequently, the new reproduction number is α*R*_0_. These preventive measures are kept until the ongoing epidemic is nearly finished. That is, all preventive measures are relaxed thus setting α back to 1 on 30 June. If herd immunity is not reached, then there will then be a second wave, whereas if herd immunity has been achieved, the epidemic continues to decline.

We used the model to investigate the effect of the preventive measures and, for two scenarios, we analyzed whether a given level of preventive measures yields disease-induced herd immunity. We did this for populations that are (i) homogeneous, (ii) categorized by age groups but not by activity levels, (iii) not categorized by age but assigned different activity levels, and (iv) have both age-related and activity structures.

For each of the four population structures described above, we show overall disease-induced herd immunity in Table 1. This was obtained by assuming that preventive measures having factor α < 1 are implemented at the start of an epidemic, running the resulting model epidemic to its conclusion and then exposing the population to a second epidemic with α = 1. We obtain α__*__, the greatest value of α such that a second epidemic cannot occur. The disease-induced herd immunity level *h*_D_ is given by the fraction of the population that is infected by the first epidemic. This approximates the situation in which preventive measures are implemented early and lifted late in an outbreak. Note that given the next-generation matrix, *h*_D_ is independent of the distributions of the latent and infectious periods.

**Table 1:** Disease-induced herd immunity level *h*_D_ and classical herd immunity level *h*_C_ for different population structures. Numbers correspond to percentages.

**Population structure**	***R*_0_ = 2.0**		***R*_0_ = 2.5**		***R*_0_ = 3.0**	
	***h*_D_**	***h*_C_**	***h*_D_**	***h*_C_**	***h*_D_**	***h*_C_**
Homogeneous	50.0	50.0	60.0	60.0	66.7	66.7
Age structure	46.0	50.0	55.8	60.0	62.5	66.7
Activity structure	37.7	50.0	46.3	60.0	52.5	66.7
Age and activity structure	34.6	50.0	43.0	60.0	49.1	66.7

As seen in Table 1, all three structured populations have lower disease-induced herd immunity *h*_D_ compared with the classical herd immunity *h*_C_, which assumes that immunity is uniformly distributed among the different types of individuals. From the table, it is clear that the different activity levels have a greater effect on reducing *h*_D_ than does age structure.

In Table 2, the final fractions infected in the different age activity groups for α = α__*__ just barely reaching disease-induced herd immunity are given. This is done for the age and activity group structure and assuming *R*_0_ = 2.5. The overall fraction infected equals *h*_D_ = 43.0%, in agreement with Table 1. Table S1 is a similar table in which only activity groups are considered.

**Table 2:** Final outcome fractions infected in different groups. These values assume that *R*_0_ = 2.5 and preventive measures are put in place such that α = α__*__ just barely reaching herd immunity for *R*_0_ = 2.5. Population structure includes both age and activity. Numbers correspond to percentages.

**Age group**	**Low activity**	**Average activity**	**High activity**
0–5 years	17.6	32.1	53.9
6–12 years	25.8	44.9	69.7
13–19 years	31.4	52.9	77.8
20–39 years	27.4	47.2	72.1
40–59 years	22.8	40.3	64.4
≥60 years	14.6	27.0	46.7

**Fig. 1:**
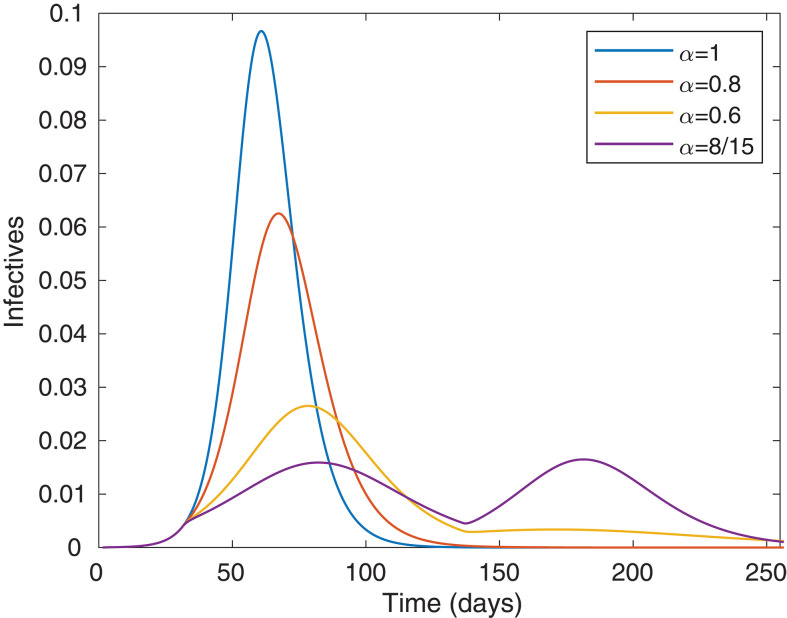
Overall fraction infected over time. Shown is a plot of the overall fraction infected over time for the age and activity structured community with *R*_0_ = 2.5 for four different preventive levels inserted 15 March (day 30) and lifted 30 June (day 135). The blue, red, yellow, and purple curves correspond to no, light, moderate, and severe preventive measures, respectively.

We also illustrate the time evolution of the epidemic for *R*_0_ = 2.5, assuming both age and activity structure and starting with a small fraction externally infected in mid-February. For this, we show the epidemic over time for four different levels of preventive measures put in place early in the epidemic outbreak (mid-March) and being relaxed once transmission has dropped to low levels (30 June). In Fig. 1, the community proportion that is infectious during the course of the epidemic is plotted.

**Fig. 2:**
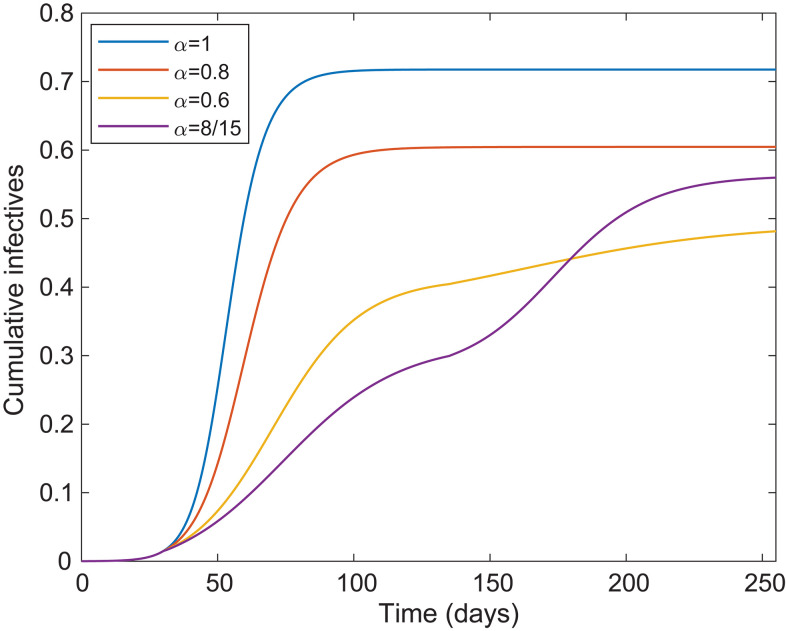
Cumulative fraction infected over time. Shown is a plot of the cumulative fraction infected over time for the age and activity structured community and *R*_0_ = 2.5 for four different preventive levels inserted 15 March (day 30) and lifted 30 June (day 135). The blue, red, yellow, and purple curves correspond to no, light, moderate, and severe preventive measures, respectively.

On 15 March, preventive measures (at four different levels for α) are put in place and in every case, the growth rate is reduced except when no preventative measures are applied (the blue curve; α = 1). Moreover, the preventive measures reduce the size of, and delay the time of, the peak. Sanctions are lifted on 30 June, putting transmission rates back to their original levels, but only in the curve with highest sanctions is there a clear second wave because the remaining curves have reached (close to) herd immunity. The yellow curve finishes at <50% getting infected. The reason this exceeds the 43% infected shown in Table 1 is that preventive measures were not imposed from the start and were lifted before the epidemic was over. The corresponding cumulative fraction infected as a function of time is shown in Fig. 2. An interesting observation is that the purple curve results in a higher overall fraction infected even though this scenario had more restrictions applied than the scenario of the yellow curve. This is because this epidemic was further from completion when sanctions were lifted.

Only the curve corresponding to greatest preventive measures shows a severe second wave when restrictions are lifted. In most cases, no (strong) second wave of outbreak occurs once preventive measures are lifted. Note also that the yellow curve, in which the overall fraction infected is well below the classical herd immunity level *h*_C_ = 60%, is in fact protected by herd immunity because no second wave appears. See the supplementary materials for depictions of when restrictions are lifted continuously between 1 June and 31 August (figs. S1 and S2) and how the effective reproduction number evolves as a function of the time when restrictions are lifted (fig. S3).

Our simple model shows how the disease-induced herd immunity level may be substantially lower than the classical herd immunity level derived from mathematical models assuming homogeneous immunization. Our application to COVID-19 indicates a reduction of herd immunity from 60% under homogeneous immunization down to 43% (assuming *R*_0_ = 2.5) in a structured population, but this should be interpreted as an illustration rather than as an exact value or even a best estimate. Future efforts need to be made to quantify more precisely the size of this effect.

In our model, we have taken age cohorts and social activity levels into account. However, more complex and realistic models have many other types of heterogeneities; for instance, increased spreading within households (of different sizes) or within schools and workplaces. These activity levels and social structures are country or region specific and should be modeled as such. Further, spatial heterogeneity arises, with rural areas having lower contact rates than metropolitan regions. It seems reasonable to assume that most such additional heterogeneities will have the effect of reducing the disease-induced immunity level *h*_D_ even further. This is because in high-contact environments such as metropolitan regions, large households, and large workplaces, there will be a higher infected fraction and immunity will be concentrated even more among highly active and connected individuals. Some complex models [e.g., (*1*)] categorize by, for example, age and spatial location but omit individual variation within each category. The latter can be incorporated by including different activity levels or by adding a social network in which individuals have differing numbers of acquaintances. As we have illustrated, differences in social activity play a greater role in reducing the disease-induced herd immunity level than heterogeneous age-group mixing. Therefore, models excluding such features will see a smaller difference between *h*_D_ and *h*_C_. Our choice of 50% having average activity, 25% having half activity, and 25% having double activity is of course arbitrary. An important future task is to determine the size of differences in social activity within age groups for different types of populations. The greater the social heterogeneity there is between groups, the greater the difference between *h*_D_ and *h*_C_.

One assumption of our model is that preventive measures act proportionally on all contact rates, and this may not always hold. For example, most countries aim to protect elderly and other high-risk groups, which does not obey this assumption. Again, we expect that the effect of discriminatory protection would be to reduce the disease-induced immunity level because the oldest age group has the fewest contacts. For a model including schools and workplaces, it is not obvious what effect school closure and strong recommendations to work from home would have on the disease-induced herd immunity level. A different model extension would be to allow individuals to change their activity levels over time. The effect of such changes in activity levels, particularly whether they vary between different categories, remains unknown.

In our model, we assume that infection with and subsequent clearance of the virus leads to immunity against further infection for an extended period of time. If there is relatively quick loss of immunity, or if we want to consider a time scale in which the impact of demographic processes such as births and people changing age groups becomes substantial, then we need further models.

Different forms of immunity levels have been discussed previously in the literature, although, as far as we know, not when considering early-introduced preventions that are lifted toward the end of an epidemic outbreak. Anderson and May (*10*) concluded that immunity level may differ among uniformly distributed, disease-induced, and optimally located immunity [see also (*11*)], and vaccination policies selecting individuals to immunize in an optimal manner have been discussed in many previous studies [e.g., (*12*)]. A recent independent study by Gomes *et al*. (*13*) reported results similar to those of the present study but considers heterogeneities in terms of continuously varying susceptibilities. That model is solved numerically in a manner similar to our Fig. 1, but the analytical results for the final number of infected people and *h*_D_ are missing.

Rather than lifting all COVID-19 preventive measures simultaneously, most countries are lifting restrictions gradually. That strategy can prevent the type of overshoot illustrated by the purple curve in Fig. 2, which results in a greater fraction infected than if milder restrictions are enacted (yellow curve).

## Supplementary Material

science.sciencemag.org/content/369/6505/846/suppl/DC1 Materials and Methods Figs. S1 to S3 Table S1 References (*15**, **16*) MDAR Reproducibility Checklist
